# Supplementary material for: The co-inhibitory receptor TIGIT promotes tissue-protective functions in T cells
Source: Nat Immunol. 2025 Oct 15;26(11):2074–85. doi: 10.1038/s41590-025-02300-w (PMC12571903; doi:10.1038/s41590-025-02300-w)
Supplement: Supplementary file 1 — Reporting Summary [file 41590_2025_2300_MOESM1_ESM.pdf]

nature portfolio

Corresponding author(s): Nicole Joller

Last updated by author(s): 2025/08/23

Reporting Summary

Nature Portfolio wishes to improve the reproducibility of the work that we publish. This form provides structure for consistency and transparency in reporting. For further information on Nature Portfolio policies, see our Editorial Policies and the Editorial Policy Checklist.

Statistics

For all statistical analyses, confirm that the following items are present in the figure legend, table legend, main text, or Methods section.

n/a	Confirmed	
☐	☒	The exact sample size (*n*) for each experimental group/condition, given as a discrete number and unit of measurement
☐	☒	A statement on whether measurements were taken from distinct samples or whether the same sample was measured repeatedly
☐	☒	The statistical test(s) used AND whether they are one- or two-sided<br>*Only common tests should be described solely by name; describe more complex techniques in the Methods section.*
☒	☐	A description of all covariates tested
☐	☒	A description of any assumptions or corrections, such as tests of normality and adjustment for multiple comparisons
☐	☒	A full description of the statistical parameters including central tendency (e.g. means) or other basic estimates (e.g. regression coefficient) AND variation (e.g. standard deviation) or associated estimates of uncertainty (e.g. confidence intervals)
☐	☒	For null hypothesis testing, the test statistic (e.g. *F*, *t*, *r*) with confidence intervals, effect sizes, degrees of freedom and *P* value noted<br>*Give P values as exact values whenever suitable.*
☒	☐	For Bayesian analysis, information on the choice of priors and Markov chain Monte Carlo settings
☒	☐	For hierarchical and complex designs, identification of the appropriate level for tests and full reporting of outcomes
☐	☒	Estimates of effect sizes (e.g. Cohen's *d*, Pearson's *r*), indicating how they were calculated

Our web collection on statistics for biologists contains articles on many of the points above.

Software and code

Policy information about availability of computer code

Data collection	The Flow cytometric assays: BD LSR Fortessa (BD Bioscience), BD FACS Symphony A5 (BD Bioscience), Cytek Aurora (Cytek Biosciences)<br>The single-cell RNA-Seq analysis: NovaSeq6000 (Illumina)
Data analysis	Digital gene expression matrix of single cell sequencing data was obtained from the raw reads using Cell Ranger (v7.0.0).<br>Seurat (v4.0.3 and v5.0.1) was used to further analyze the data.<br>The R package DoubletFinder (v2.0) was applied to filter doublet cells. Batch correction was performed using Harmony (v1.2). SCENIC (v1.1.2) was used to identify regulons. Visualizations were performed with ggplot2 (v3.5.1). Pearson r correlation was obtained from ggpubr (v0.6.0).<br>The code is available at https://github.com/hemberg-lab/tigit-paper-figures<br>The integrated transcriptome and TCR repertoire data object was generated using the R package Platypus (v3.1). Heatmaps displaying clonal overlap were produced using the pheatmap package (v1.0.12).<br>Statistical analysis: GraphPad Prism (v10.0.3)<br>Flow cytometric analysis: FlowJo (v10.8.2 and 10.10.0), SpectroFlo (Cytek Biosciences, v3.0.3) ,

For manuscripts utilizing custom algorithms or software that are central to the research but not yet described in published literature, software must be made available to editors and reviewers. We strongly encourage code deposition in a community repository (e.g. GitHub). See the Nature Portfolio guidelines for submitting code & software for further information.

Data

Policy information about availability of data

All manuscripts must include a data availability statement. This statement should provide the following information, where applicable:

- Accession codes, unique identifiers, or web links for publicly available datasets
- A description of any restrictions on data availability
- For clinical datasets or third party data, please ensure that the statement adheres to our policy

All scRNA-seq and scTCR-seq data from WT and TIGIT KO T cells have been deposited on the ArrayExpress database at EMBL-EBI (www.ebi.ac.uk/arrayexpress) and is available via accession number E-MTAB-8861, the Seurat Object was deposited at Zenodo and is available via doi: 10.5281/zenodo.14041419.
Blimp-1 ChIP-seq and RNA-seq data was previously reported and is available in the Gene Expression Omnibus database under accession numbers GSE79339 and GSE121838.

Research involving human participants, their data, or biological material

Policy information about studies with human participants or human data. See also policy information about sex, gender (identity/presentation), and sexual orientation and race, ethnicity and racism.

Reporting on sex and gender	not applicable
Reporting on race, ethnicity, or other socially relevant groupings	not applicable
Population characteristics	not applicable
Recruitment	not applicable
Ethics oversight	not applicable

Note that full information on the approval of the study protocol must also be provided in the manuscript.

Field-specific reporting

Please select the one below that is the best fit for your research. If you are not sure, read the appropriate sections before making your selection.

☒ Life sciences ☐ Behavioural & social sciences ☐ Ecological, evolutionary & environmental sciences

For a reference copy of the document with all sections, see nature.com/documents/nr-reporting-summary-flat.pdf

Life sciences study design

All studies must disclose on these points even when the disclosure is negative.

Sample size	Sample sizes were chosen based on power calculations using estimated effect size ranges that were based on our previous experience. When additional datapoints were collected under identical conditions in later experiments with, these were included in the pooled data. Sample size was sufficient to demonstrate statistically significant differences in comparisons between experimental groups by the respective statistical tests as indicated in the figure legends. Adequate sample size was confirmed based on reproducibility between independent experiments.
Data exclusions	Wherever possible, experiments included a positive and negative control. If results obtained for these controls were not as expected, data points were excluded.
Replication	All experiments were repeated at least twice to confirm reproducibility, with specific sample sizes and details indicated in figure captions.
Randomization	For animal experiments, animals with the same genotype were randomized to the the different treatment groups.
Blinding	For histological analysis, samples were blinded.<br>Due to technical reasons (indication of genotypes and treatment on cage cards of animals) animal experiments were not carried out in a blinded manner.

Reporting for specific materials, systems and methods

We require information from authors about some types of materials, experimental systems and methods used in many studies. Here, indicate whether each material, system or method listed is relevant to your study. If you are not sure if a list item applies to your research, read the appropriate section before selecting a response.

Materials & experimental systems

n/a	Involved in the study
☐	☒ Antibodies
☒	☐ Eukaryotic cell lines
☒	☐ Palaeontology and archaeology
☐	☒ Animals and other organisms
☒	☐ Clinical data
☒	☐ Dual use research of concern
☒	☐ Plants

Methods

n/a	Involved in the study
☒	☐ ChIP-seq
☐	☒ Flow cytometry
☒	☐ MRI-based neuroimaging

Antibodies

Antibodies used

Antibody, Fluorochrome, Dilution factor, Clone, Supplier:
Areg Alexa-Fluor647 1:100 G-4 Santa Cruz
Areg Biotin 1:200 - R&D
B220 BUV563 1:200 RA3-6B2 BD
Blimp-1 APC 1:100 5E7 BioLegend
Blimp-1 BV421 1:100 5E7 BD
CD112 BV711 1:100 829038 BD
CD11b PerCP 1:400 M1/70 BioLegend
CD11c BV605 1:100 N418 BioLegend
CD155 BUV737 1:100 TX56 BD
CD155 PE 1:300 TX56 BioLegend
CD226 APC/Fire750 1:100 10E5 BioLegend
CD226 PE-Cy7 1:100 10E5 BD
CD3 BUV805 1:100 17A2 eBioscience
CD3 BV785 1:200 17A2 BioLegend
CD4 BUV737 1:300 RM4-5 BD
CD4 APC 1:200 RM4-5 BioLegend
CD4 BUV496 1:500 RM4-5 BD
CD4 APC-Cy7 1:300 GK1.5 BD
CD44 APC/Fire750 1:300 IM7 BioLegend
CD45 BUV805 1:300 30-F11 BD
CD62L BV650 1:400 MEL-14 BioLegend
CD8 BUV395 1:500 53-6.7 BD
CXCR3 Biotin 1:100 SA051D1 BioLegend
F4/80 APC-Fire810 1:100 BM8 BioLegend
Foxp3 FITC 1:200 FJK-16s eBioscience
Foxp3 PE 1:200 FJK-16s Thermo
Foxp3 eF450 1:200 FJK-16s eBioscience
Gata3 PE-Cy5 1:200 TWAJ eBioscience
GM-CSF PE-Cy7 1:100 MP1-22E9 BioLegend
Granzyme B APC 1:100 GB12 Thermo
GFP Tag AF488 1:400 FM264G BioLegend
Helios BUV563 1:100 22F6 eBioscience
IFN-g PE 1:300 XMG1.2 BioLegend
JunB CoraLitePlus488 1:125 - ProteinTech
Ki-67 BUV737 1:300 SolA15 Thermo
Klrg1 BUV661 1:200 2F1 BD
LD Zombie NIR 1:500 - BioLegend
LD Blue 1:500 - Thermo
Ly6G BV750 1:200 1A8 BD
NK1.1 BUV615 1:200 PK136 BD
Nrp1 BV7711 1:500 V46-1954 BD
pAKT AF647 1:10 M89-61 BD
PD-1 BV785 1:500 29F.1A12 BioLegend
pS6 PerCP-eF710 1:10 cupk43k eBioscience
ST2 Biotin 1:100 DIH9 BioLegend
Streptavidin BUV563 1:800 - eBioscience
Streptavidin BV711 1:300 - BioLegend
Streptavidin BV480 1:500 - BD
TCF1/7 R718 1:100 S33-966 BD
TCRb PE-Dazzle594 1:300 H57-597 BioLegend
TCRgd BB700 1:200 GL3 BD
TIGIT PE-Dazzle594 1:50 1G9 BioLegend
TIGIT BV421 1:50 TX99 BD
TIGIT PerCP-eF710 1:200 GIGD7 Thermo
TNF-a BV421 1:100 MP6-XT22 BioLegend

Validation	All antibodies used in this study were validated for species and application by the vendors. All antibodies were further validated and titrated using isotype (and where possible biological) control samples in each experiment.

Animals and other research organisms

Policy information about studies involving animals; ARRIVE guidelines recommended for reporting animal research, and Sex and Gender in Research

Laboratory animals	Adult 7-45 weeks old male and female mice were used and age and sex were matched between experimental groups.<br>The following strains were used:<br>C57BL/6 (from Janvier)<br>Foxp3-GFP.KI (from VK Kuchroo)<br>Tigitfl/fl x B6.129(Cg)-Foxp3tm4(YFP/icre)Ayr/J (from VK Kuchroo)<br>Tigitfl/fl x Foxp3tm9(EGFP/cre/ERT2)Ayr/J (crossed in Zurich, parental strains from VK Kuchroo and JAX)<br>Tigitfl/fl x C57BL/6-Tg(Cd8a-cre)1Itan/J (crossed in Zurich, parental strains from VK Kuchroo and JAX)<br>B6.Cg-Tg(Cd4-cre)1Cwi/BfluJ x B6.129-Hif1atm3Rsjo/J (from N Aceto)<br>Lckcre x Prdm1fl/fl (from JAX)<br>B6N.129S2-Pvr<tm1Gbn>/J (from T Korn)<br>LckcrexPrdm1fl/fl mice were bred and housed at the Peter Doherty Institute, Melbourne, Australia, all other strains at the University of Zurich or the University of Basel, Switzerland, under specific pathogen-free conditions. Animals were housed in individually ventilated cages containing autoclaved bedding and nesting material, with standard diet and water ad libitum and a 12h light/dark cycle (18–23°C, 40-60% humidity).
Wild animals	not applicable
Reporting on sex	Male and female mice were used and sex was matched between experimental groups.<br>For experiments using heterozygous Tigitfl/fl x Foxp3tm9(EGFP/cre/ERT2)Ayr/J mice, females were used as the Foxp3 gene is located on the x chromosome.
Field-collected samples	not applicable
Ethics oversight	Cantonal veterinary office of Zurich<br>Cantonal veterinary office of Basel<br>University of Melbourne animal ethics committee

Note that full information on the approval of the study protocol must also be provided in the manuscript.

Plants

Seed stocks	not applicable
Novel plant genotypes	not applicable
Authentication	not applicable

Flow Cytometry

Plots

Confirm that:

- [x] The axis labels state the marker and fluorochrome used (e.g. CD4-FITC).
- [x] The axis scales are clearly visible. Include numbers along axes only for bottom left plot of group (a 'group' is an analysis of identical markers).
- [x] All plots are contour plots with outliers or pseudocolor plots.
- [x] A numerical value for number of cells or percentage (with statistics) is provided.

Methodology

Sample preparation	Single-cell suspensions of spleen were generated by mechanical disruption in RPMI 1640 (Gibco) supplemented with 10% FCS (Corning or Capri), penicillin and streptomycin (100 U/ml, Gibco), 2mmol/l L-glutamine (Gibco), Brefeldin A (BioLegend) and Marimastat (Sigma). Red blood cells were lysed using ACK lysis buffer (155 mM NH4Cl, 10 mM KHCO3, 0.1 mM Na2EDTA, pH: 7,4) for 3-5 minutes. Single-cell suspensions of the lung were generated by enzymatic tissue digestion at 37 °C with 0.2 mg/

ml Deoxyribonuclease I (Sigma) and 2.4 mg/ml Collagenase type I (Gibco) and mechanical disruption using the GentleMACS. Media was supplemented with Brefeldin A (BioLegend) and Marimastat (Sigma). Lung immune cells were separated from epithelial and parenchymal cells by 30% Percoll gradient centrifugation (GE Healthcare).
Single-cell suspensions were directly stained ex vivo or incubated at 37°C for 2h 45min with Brefeldin A (BioLegend), Monensin (BioLegend) and Marimastat (Sigma). Samples were incubated with surface antibodies diluted in PBS for 20 minutes at RT. For intracellular cytokines staining, cells were permeabilized using the Cytofix/Cytoperm kit (BD Bioscience) for 5-10 minutes at RT, followed by 30 minutes incubation at RT with the intracellular antibodies mix. For transcription factors staining, cells were permeabilized with the Foxp3/Transcription factor staining buffer set (eBioscience) for 40 minutes at RT. The Zombie NIR or LD Blue fixable dyes were used to exclude dead cells.

Instrument

BD LSR Fortessa (BD Bioscience), BD FACS Symphony A5 (BD Bioscience), Cytek Aurora (Cytek Biosciences)

Software

SpectroFlo (Cytek Biosciences, v3.0.3) and FlowJo (BD Bioscience, v10.8.2 or 10.10.0) software were used for data analysis.. For the R analysis: unmixed and pre-gated cells were imported into RStudio using R (v4.4.1) and the flowCore (v2.16.0) and CATALYST (v1.28.0) packages. Data was analyzed in the FlowJo workspace using the flowWorkspace (v4.16.0) and CytoML (v2.16.0) packages and the UMAP and FlowSOM plugins.

Cell population abundance

not applicable

Gating strategy

Cells cells were first gated by FSC/SSC to exclude debris, followed by gating by FSC-A/FSC-H to exclude doublets and by LiveDead dye/CD45 to exclude dead cells. Target cell populations for further analysis were gated by cell lineage markers (CD4, CD8, Foxp3). Gates for all subsequent markers were set based on isotype controls. The gating strategy is illustrated in Extended Data Figure 10.

☒ Tick this box to confirm that a figure exemplifying the gating strategy is provided in the Supplementary Information.
